# Supplementary figures and images for: Cryptic functional diversity within a grass mycobiome
Source: PLoS One. 2023 Jul 20;18(7):e0287990. doi: 10.1371/journal.pone.0287990 (PMC10358963; doi:10.1371/journal.pone.0287990)

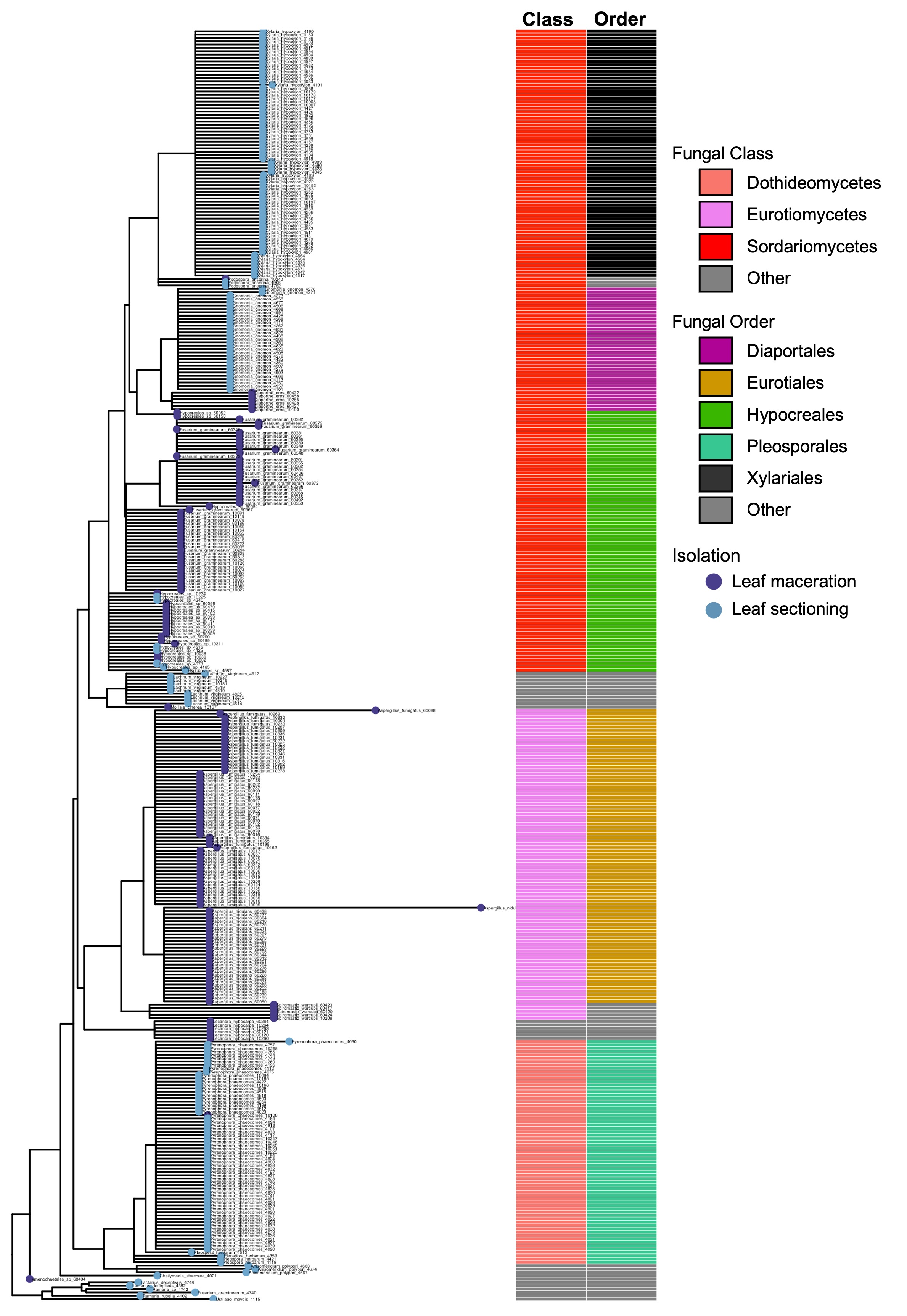

Supplement: S1 Fig — Species names assigned to isolate are indicated at the tips of tree and are color-coded by isolation method (sky-blue: leaf sectioning, dark purple: leaf maceration). Adjacent columns represent fungal class and order (“Other” represents fungal classes or orders with a relative abundance ≤ 2%). (JPG) [file pone.0287990.s001.jpg]

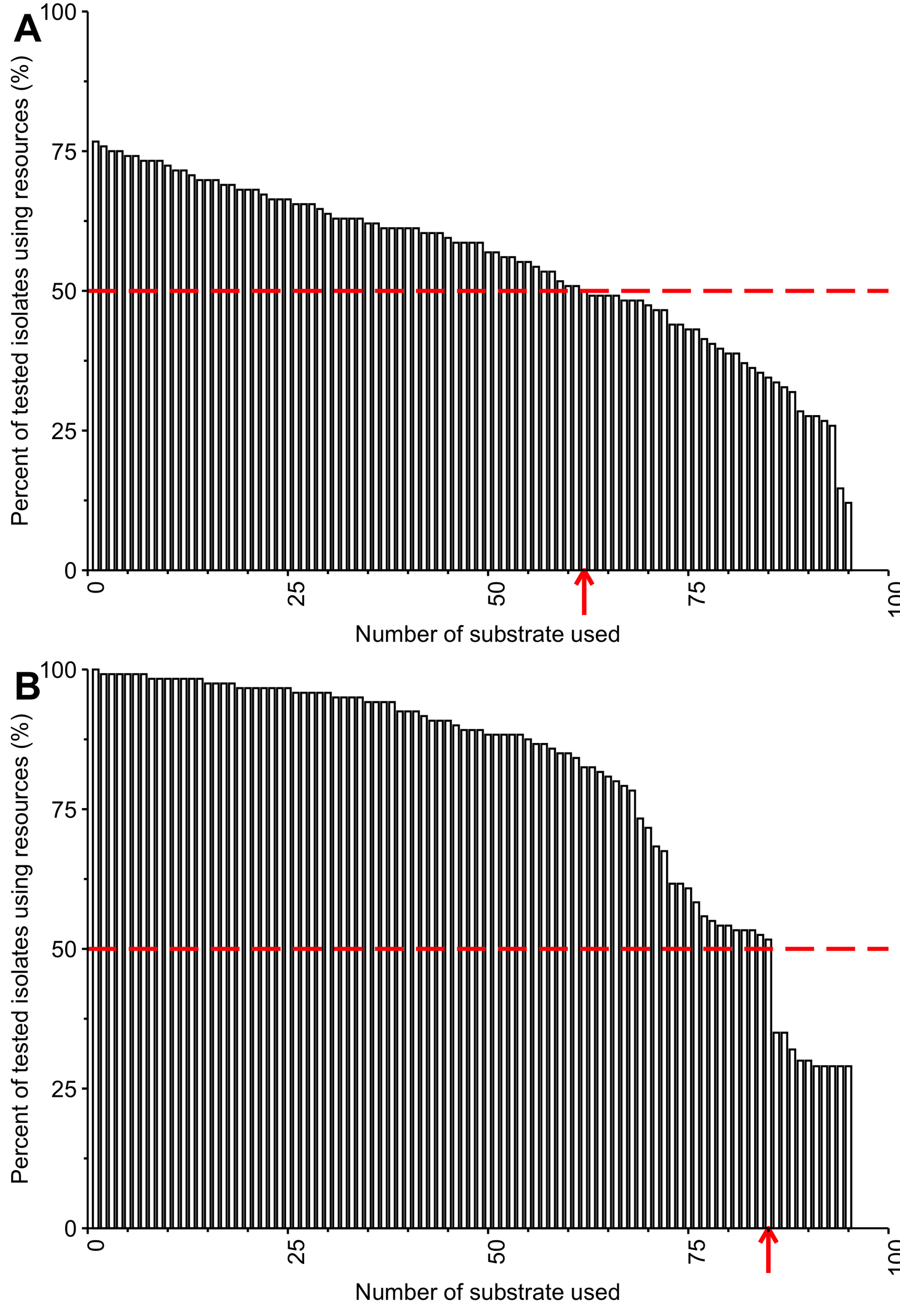

Supplement: S2 Fig — Graph shows the percent of fungal isolates that use (standardized growth > 0.005 OD590) a given number of Biolog carbon resources. Of the 240 isolates evaluated for use and growth on Biolog carbon resources, 50% of isolates (red dotted line) obtained by leaf sectioning (A) used 62 Biolog carbon resources (red arrow). For isolates obtained by leaf maceration (B), 50% used 85 Biolog carbon resources. (TIF) [file pone.0287990.s002.tif]

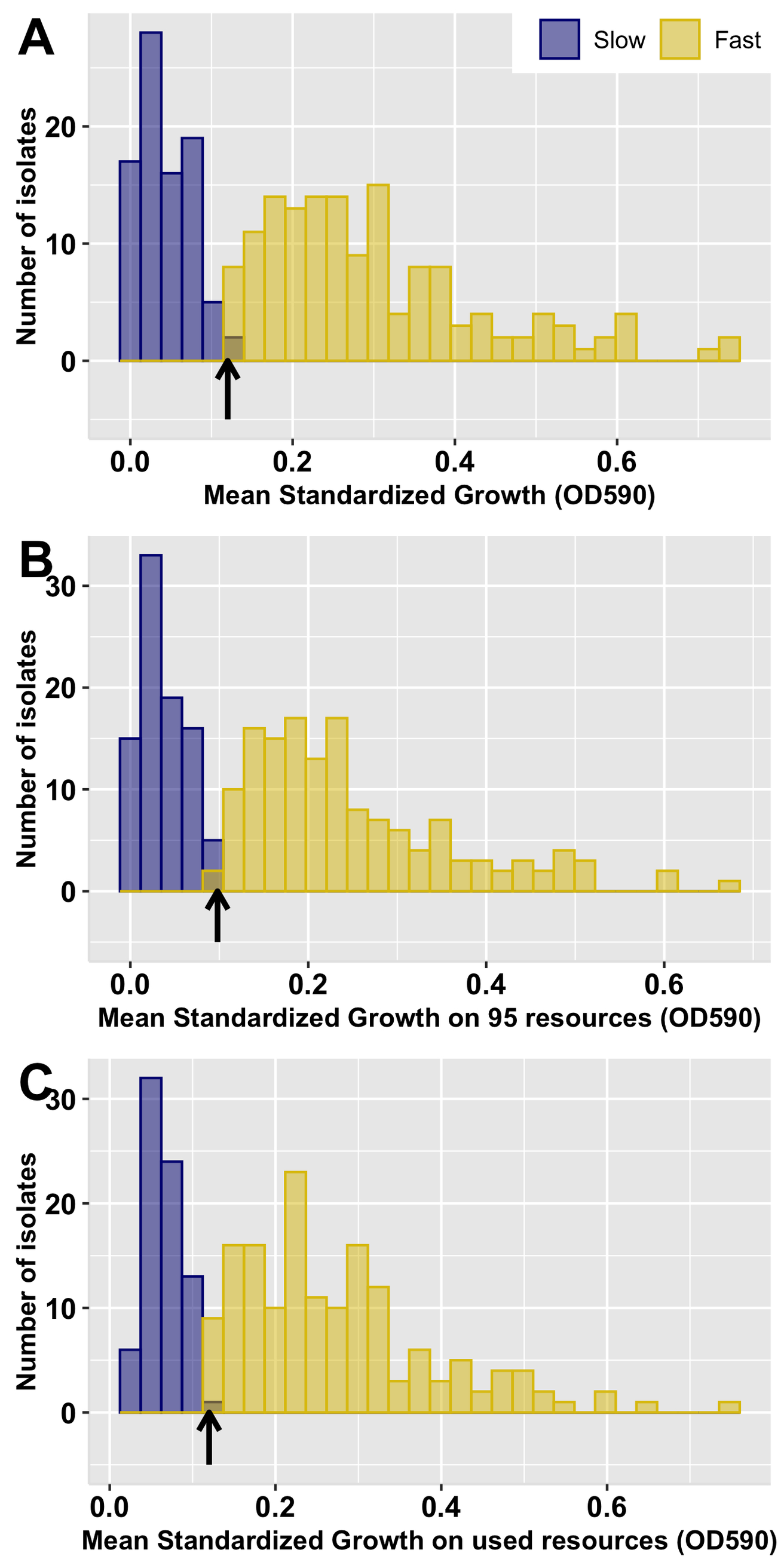

Supplement: S3 Fig — Mean standardized growth of fungal isolates calculated using three different metrics demonstrated bimodal distributions. Mean standardized growth was calculated on the basis of 62 Biolog carbon resources (A), all 95 resources (B) and only those resources used by each individual isolate (C). Cut-off values derived from finite mixture model fitting ([87], A; 0.12, B; 0.12, C; 0.1) distinguishing slow- (blue bars) and fast-growing (gold bars) assemblage are shown (black arrow). (TIF) [file pone.0287990.s003.tif]
