# Supplementary material for: Cryptic functional diversity within a grass mycobiome
Source: PLoS One. 2023 Jul 20;18(7):e0287990. doi: 10.1371/journal.pone.0287990 (PMC10358963; doi:10.1371/journal.pone.0287990)
Supplement: S1 Table — For each of the 95 Biolog carbon resources, the percent of isolates obtained by each isolation method and using that resource are indicated. Resources are rank in order from most often used (1) to least often used resources by both sets of isolates. Mean standardized growth of each isolate was calculated based on standardized growth on the 62 resources used by at least 50% of the isolates (ranks in bold). (DOCX) [file pone.0287990.s004.docx]

| **Rank** | **Biolog carbon resource** | **Percent of isolates using resource** | | **Rank** | **Biolog carbon resource** | **Percent of isolates using resource** | |
| --- | --- | --- | --- | --- | --- | --- | --- |
|  |  | **Sectioning** | **Maceration** |  |  | **Sectioning** | **Maceration** |
| **1** | Maltotriose | 77 | 100 | **49** | Succinic Acid | 60 | 85 |
| **2** | D-Raffinose | 76 | 98 | **50** | D-Lactic Acid Methyl Ester | 59 | 86 |
| **3** | D-Melezitose | 75 | 98 | **51** | Uridine | 55 | 87 |
| **4** | D-Mannose | 73 | 100 | **52** | Thymidine-5'-Monophosphate | 61 | 80 |
| **5** | Lactulose | 74 | 98 | **53** | Inulin | 56 | 78 |
| **6** | D-Mannitol | 73 | 99 | **54** | p-Hydroxy-Phenylacetic Acid | 51 | 81 |
| **7** | D-Melibiose | 73 | 97 | **55** | Thymidine | 69 | 62 |
| **8** | Turanose | 72 | 98 | **56** | 2-Deoxy Adenosine | 59 | 72 |
| **9** | D-Xylose | 69 | 100 | **57** | D-Alanine | 61 | 56 |
| **10** | Stachyose | 72 | 96 | **58** | 2,3-Butanediol | 54 | 61 |
| **11** | D-Fructose | 70 | 98 | **59** | Uridine-5'-Monophosphate | 61 | 53 |
| **12** | L-Rhamnose | 72 | 95 | **60** | Adenosine | 60 | 54 |
| **13** | Palatinose | 68 | 99 | **61** | alpha-D-Glucose-1-Phosphate | 53 | 53 |
| **14** | Maltose | 68 | 98 | **62** | beta-Cyclodextrin | 50 | 54 |
| **15** | D-Sorbitol | 67 | 99 | 63 | L-Pyroglutamic Acid | 48 | 97 |
| **16** | alpha-D-Glucose | 66 | 100 | 64 | Amygdalin | 47 | 98 |
| **17** | Gentiobiose | 66 | 100 | 65 | Tween 80 | 47 | 98 |
| **18** | D-Cellobiose | 66 | 100 | 66 | L-Alanyl-Glycine | 47 | 98 |
| **19** | D-Trehalose | 66 | 100 | 67 | N-Acetyl-beta-D-Mannosamine | 49 | 93 |
| **20** | Salicin | 74 | 91 | 68 | D-Arabitol | 43 | 98 |
| **21** | alpha-D-Lactose | 68 | 97 | 69 | L-Glutamic Acid | 49 | 92 |
| **22** | beta-Methyl-D-Glucoside | 70 | 94 | 70 | alpha-Methyl-D-Glucoside | 44 | 97 |
| **23** | L-Alanine | 66 | 97 | 71 | Glycerol | 41 | 98 |
| **24** | L-Arabinose | 62 | 100 | 72 | beta-Hydroxybutiric Acid | 49 | 89 |
| **25** | D-Galacturonic Acid | 65 | 95 | 73 | Inosine | 49 | 88 |
| **26** | Arbutin | 64 | 96 | 74 | Succinic Acid Mono-Methyl Ester | 48 | 89 |
| **27** | D-Ribose | 60 | 99 | 75 | m-Inositol | 37 | 95 |
| **28** | D-Galactose | 61 | 97 | 76 | alpha-Ketoglutaric Acid | 35 | 96 |
| **29** | N-Acetyl-D-Glucosamine | 59 | 99 | 77 | Glycyl-L-Glutamic Acid | 43 | 88 |
| **30** | Lactamide | 75 | 83 | 78 | L-Lactic Acid | 40 | 91 |
| **31** | gamma-Hydroxybutiric Acid | 63 | 94 | 79 | Succi0mic Acid | 39 | 88 |
| **32** | L-Fucose | 61 | 96 | 80 | alpha-Hydroxybutiric Acid | 34 | 88 |
| **33** | Tween 40 | 57 | 100 | 81 | Acetic Acid | 26 | 95 |
| **34** | alpha-Methyl-D-Mannoside | 63 | 93 | 82 | Propionic Acid | 32 | 88 |
| **35** | Xylitol | 63 | 93 | 83 | Putrescine | 33 | 87 |
| **36** | Man0n | 62 | 94 | 84 | L-Alani0mide | 34 | 83 |
| **37** | D-Psicose | 70 | 85 | 85 | 3-Methyl-D-Glucose | 48 | 68 |
| **38** | Dextrin | 55 | 100 | 86 | Pyruvic Acid Methyl Ester | 28 | 79 |
| **39** | D-Tagatose | 66 | 88 | 87 | L-Malic Acid | 36 | 68 |
| **40** | beta-Methyl-D-Galactoside | 71 | 82 | 88 | D-Fructose-6-Phosphate | 41 | 62 |
| **41** | L-Serine | 57 | 94 | 89 | alpha-Ketovaleric Acid | 28 | 73 |
| **42** | Sucrose | 52 | 99 | 90 | alpha-Cyclodextrin | 44 | 55 |
| **43** | L-Asparagine | 53 | 97 | 91 | D-Glucose-6-Phosphate | 39 | 52 |
| **44** | Pyruvic Acid | 59 | 90 | 92 | Adenosine-5'-Monophosphate | 28 | 53 |
| **45** | Glycogen | 59 | 89 | 93 | N-Acetyl-L-Glutamic Acid | 15 | 58 |
| **46** | alpha-Methyl-D-Galactoside | 63 | 84 | 94 | D-Malic Acid | 12 | 53 |
| **47** | D-Gluconic Acid | 51 | 96 | 95 | D-L-alpha-Glycerol Phosphate | 27 | 35 |
| **48** | Sedoheptulosan | 56 | 90 |  |  |  |  |
