# Supplementary material for: Cryptic functional diversity within a grass mycobiome
Source: PLoS One. 2023 Jul 20;18(7):e0287990. doi: 10.1371/journal.pone.0287990 (PMC10358963; doi:10.1371/journal.pone.0287990)
Supplement: S2 Table — For each OTU, the assemblage (fast, slow, or shared in both assemblages), Phylum, Class, Order, and Genus/species of best taxon matches are reported. GenBank Accession numbers of reference sequences used to assign taxonomy using the Evolutionary Placement Approach (EPA) within TBAS [77, 78] are given in the last column. (DOCX) [file pone.0287990.s005.docx]

| OTU ID | Assemblage | Phylum | Class | Order | Genus/species  best EPA match | GenBank accession no. reference taxa |
| --- | --- | --- | --- | --- | --- | --- |
| 7 | Slow | Ascomycota | Sordariomycetes | Xylariales | *Xylaria hypoxylon* | DQ491487 |
| 8 | Slow | Ascomycota | Sordariomycetes | Xylariales | *Xylaria hypoxylon* | DQ491487 |
| 9 | Shared | Ascomycota | Sordariomycetes | Xylariales | *Xylaria hypoxylon* | DQ491487 |
| 12 | Shared | Ascomycota | Sordariomycetes | Xylariales | *Xylaria hypoxylon* | DQ491487 |
| 17 | Fast | Ascomycota | Sordariomycetes | Xylariales | *Xylaria hypoxylon* | DQ491487 |
| 22 | Shared | Ascomycota | Sordariomycetes | Xylariales | *Xylaria hypoxylon* | DQ491487 |
| 25 | Slow | Ascomycota | Sordariomycetes | Xylariales | *Xylaria hypoxylon* | DQ491487 |
| 26 | Slow | Ascomycota | Sordariomycetes | Xylariales | *Xylaria hypoxylon* | DQ491487 |
| 32 | Slow | Ascomycota | Sordariomycetes | Xylariales | *Xylaria hypoxylon* | DQ491487 |
| 36 | Slow | Ascomycota | Sordariomycetes | Xylariales | *Xylaria hypoxylon* | DQ491487 |
| 46 | Slow | Ascomycota | Sordariomycetes | Xylariales | *Xylaria hypoxylon* | DQ491487 |
| 44 | Fast | Ascomycota | Sordariomycetes | Sordariales | *Podospora anserina* | AF388930 |
| 70 | Fast | Ascomycota | Sordariomycetes | Sordariales | *Podospora anserina* | AF388930 |
| 10 | Slow | Ascomycota | Sordariomycetes | Diaporthales | *Gnomonia gnomon* | DQ491518 |
| 23 | Slow | Ascomycota | Sordariomycetes | Diaporthales | *Gnomonia gnomon* | DQ491518 |
| 80 | Fast | Ascomycota | Sordariomycetes | Diaporthales | *Diaporthe eres* | DQ491514 |
| 65 | Fast | Ascomycota | Sordariomycetes | Hypocreales | *Fusarium graminearum* | AF132798 |
| 78 | Fast | Ascomycota | Sordariomycetes | Hypocreales | *Fusarium graminearum* | AF132798 |
| 79 | Fast | Ascomycota | Sordariomycetes | Hypocreales | *Fusarium graminearum* | AF132798 |
| 101 | Fast | Ascomycota | Sordariomycetes | Hypocreales | *Fusarium graminearum* | AF132798 |
| 102 | Fast | Ascomycota | Sordariomycetes | Hypocreales | *Fusarium graminearum* | AF132798 |
| 16 | Slow | Ascomycota | Sordariomycetes | Hypocreales | Hypocreales sp. | DQ491484 |
| 24 | Slow | Ascomycota | Sordariomycetes | Hypocreales | Hypocreales sp. | DQ491484 |
| 28 | Slow | Ascomycota | Sordariomycetes | Hypocreales | Hypocreales sp. | DQ491484 |
| 40 | Slow | Ascomycota | Sordariomycetes | Hypocreales | Hypocreales sp. | DQ491484 |
| 75 | Fast | Ascomycota | Sordariomycetes | Hypocreales | Hypocreales sp. | DQ491484 |
| 94 | Fast | Ascomycota | Sordariomycetes | Hypocreales | Hypocreales sp. | DQ491484 |
| 96 | Fast | Ascomycota | Sordariomycetes | Hypocreales | Hypocreales sp. | DQ491484 |
| 100 | Fast | Ascomycota | Sordariomycetes | Hypocreales | Hypocreales sp. | DQ491484 |
| 29 | Shared | Ascomycota | Leotiomycetes | Helotiales | *Lachnum virgineum* | DQ491485 |
| 31 | Slow | Ascomycota | Leotiomycetes | Helotiales | *Lachnum virgineum* | DQ491485 |
| 47 | Slow | Ascomycota | Leotiomycetes | Helotiales | *Lachnum virgineum* | DQ491485 |
| 76 | Fast | Ascomycota | Eurotiomycetes | Eurotiales | *Aspergillus fumigatus* | AY373851 |
| 77 | Fast | Ascomycota | Eurotiomycetes | Eurotiales | *Aspergillus fumigatus* | AY373851 |
| 83 | Fast | Ascomycota | Eurotiomycetes | Eurotiales | *Aspergillus fumigatus* | AY373851 |
| 84 | Fast | Ascomycota | Eurotiomycetes | Eurotiales | *Aspergillus fumigatus* | AY373851 |
| 88 | Fast | Ascomycota | Eurotiomycetes | Eurotiales | *Aspergillus fumigatus* | AY373851 |
| 91 | Fast | Ascomycota | Eurotiomycetes | Eurotiales | *Aspergillus fumigatus* | AY373851 |
| 92 | Fast | Ascomycota | Eurotiomycetes | Eurotiales | *Aspergillus fumigatus* | AY373851 |
| 93 | Fast | Ascomycota | Eurotiomycetes | Eurotiales | *Aspergillus fumigatus* | AY373851 |
| 98 | Fast | Ascomycota | Eurotiomycetes | Eurotiales | *Aspergillus fumigatus* | AY373851 |
| 97 | Fast | Ascomycota | Eurotiomycetes | Eurotiales | *Aspergillus nidulans* | AY373888 |
| 85 | Fast | Ascomycota | Eurotiomycetes | Onygenales | *Spiromastix warcupii* | DQ782848 |
| 90 | Fast | Ascomycota | Lecanoromycetes | Lecanorales | *Lecanora hybocarpa* | DQ782849 |
| 86 | Fast | Ascomycota | Lecanoromycetes | Lecanorales | *Hypocenomyce scalaris* | DQ782852 |
| 0 | Shared | Ascomycota | Dothideomycetes | Pleosporales | *Pyrenophora phaeocomes* | DQ491507 |
| 2 | Fast | Ascomycota | Dothideomycetes | Pleosporales | *Pyrenophora phaeocomes* | DQ491507 |
| 3 | Slow | Ascomycota | Dothideomycetes | Pleosporales | *Pyrenophora phaeocomes* | DQ491507 |
| 13 | Fast | Ascomycota | Dothideomycetes | Pleosporales | *Pyrenophora phaeocomes* | DQ491507 |
| 20 | Fast | Ascomycota | Dothideomycetes | Pleosporales | *Pyrenophora phaeocomes* | DQ491507 |
| 21 | Slow | Ascomycota | Dothideomycetes | Pleosporales | *Pyrenophora phaeocomes* | DQ491507 |
| 39 | Fast | Ascomycota | Dothideomycetes | Pleosporales | *Pyrenophora phaeocomes* | DQ491507 |
| 45 | Fast | Ascomycota | Dothideomycetes | Pleosporales | *Pyrenophora phaeocomes* | DQ491507 |
| 48 | Fast | Ascomycota | Dothideomycetes | Pleosporales | *Pyrenophora phaeocomes* | DQ491507 |
| 15 | Slow | Ascomycota | Dothideomycetes | Pleosporales | *Pleospora herbarum* | DQ491516 |
| 27 | Slow | Ascomycota | Dothideomycetes | Pleosporales | *Pleospora herbarum* | DQ491516 |
| 37 | Slow | Ascomycota | Dothideomycetes | Monoblastiales | *Anisomeridium polypori* | DQ782838 |
| 35 | Slow | Basidiomycota | Agaricomycetes | Russulales | *Lactarius deceptivus* | AY854089 |
| 41 | Slow | Basidiomycota | Agaricomycetes | Hymenochaetales | *Fomitiporia mediterranea* | AY854080 |
| 11 | Slow | Basidiomycota | Agaricomycetes | Unidentified | Unidentified | AY854078 |
| 14 | Fast | Basidiomycota | Ustilaginomycetes | Ustilaginales | *Ustilago maydis* | AY854090 |
