# Supplementary material for: Cryptic functional diversity within a grass mycobiome
Source: PLoS One. 2023 Jul 20;18(7):e0287990. doi: 10.1371/journal.pone.0287990 (PMC10358963; doi:10.1371/journal.pone.0287990)
Supplement: S3 Table — For each fungal class or order, the number of isolates that belong to either the fast- (mean standardized growth > 0.12 OD590) or slow-growing (mean standardized growth < 0.12 OD590) assemblage, and totals, are given. Equality of the number of slow- and fast-growing isolates assigned to each taxonomic group was evaluated using a pairwise Fisher exact test (NS, not significant at P < 0.05; ***: P < 0.001). Pairwise comparisons could not be made for groups with n ≤ 5 (NA). P-values were adjusted for multiple comparison using the Holm–Bonferroni method (Adjusted p-value; rowwise_fisher_test function of the rstatix package [88]). (DOCX) [file pone.0287990.s006.docx]

| **Fungal class/order** | **Slow** | **Fast** | **Total** | **Adjusted p-value** |
| --- | --- | --- | --- | --- |
| **Sordariomycetes** | 74 | 53 | 127 | ******* |
| Diaporthales | 18 | 4 | 22 | ******* |
| Hypocreales | 7 | 42 | 49 | ******* |
| Sordariales | 1 | 2 | 3 | NA |
| Xylariales | 48 | 5 | 53 | ******* |
| **Eurotiomycetes** | 1 | 57 | 58 | ******* |
| Eurotiales | 1 | 55 | 56 | ******* |
| Onygenales | 0 | 2 | 2 | NA |
| **Dothideomycetes** | 13 | 29 | 42 | 1.00 |
| Pleosporales | 12 | 29 | 41 | 1.00 |
| Unidentified  Dothideomycetes | 1 | 0 | 1 | NA |
| **Leotiomycetes** | 3 | 2 | 5 | NA |
| Helotiales | 3 | 2 | 5 | NA |
| **Lecanoromycetes** | 0 | 4 | 4 | NA |
| Lecanorales | 0 | 4 | 4 | NA |
| **Agaricomycetes** | 3 | 0 | 3 | NA |
| Aphyllophorales | 1 | 0 | 1 | NA |
| Hymeno-chaetales | 1 | 0 | 1 | NA |
| Russulales | 1 | 0 | 1 | NA |
| **Ustilaginomycetes** | 0 | 1 | 1 | NA |
| Ustilaginales | 0 | 1 | 1 | NA |
