# Supplementary material for: Cryptic functional diversity within a grass mycobiome
Source: PLoS One. 2023 Jul 20;18(7):e0287990. doi: 10.1371/journal.pone.0287990 (PMC10358963; doi:10.1371/journal.pone.0287990)
Supplement: S4 Table — Conservation of standardized growth on each of the 95 Biolog carbon resource was assessed using Blomberg’s K statistic [61] under the Brownian motion (BM) and Random Tip (RT) models and estimated for the entire phylogeny, and for the most common fungal classes, and most common orders within the class Sordariomycetes in the sample. Under the BM model, K statistics greater than 1 (K > 1; bold) indicates that traits are more conserved than expected by chance. Under the RT model, *P < 0.05 and **P < 0.01 indicates K significantly greater than expected by chance. Total number of resources for which standardized growth was more conserved than expected under null BM or RT models are reported on the last rows and these values are reported in Table 1. (DOCX) [file pone.0287990.s007.docx]

|  |  | |  | | | **Fungal class** | |  | | |  | | **Fungal order** | |  | |  |
| --- | --- | --- | --- | --- | --- | --- | --- | --- | --- | --- | --- | --- | --- | --- | --- | --- | --- |
|  | Entire Phylogeny | | Dothideomycetes | | | Eurotiomycetes | | Sordariomycetes | | | Hypocreales | | Xylariales | | Diaportales | |  |
| Trait | K_obs_ | *P* | K_obs_ | *P* | K_obs_ | | *P* | | K_obs_ | *P* | K_obs_ | *P* | K_obs_ | *P* | K_obs_ | *P* |  |
| 2,3-Butanediol | 0.40 | 0.67 | 0.97 | 0.18 | 0.77 | | 0.35 | | 0.83 | 0.46 | 0.92 | 0.29 | 0.87 | 0.20 | 0.82 | 0.47 |  |
| 2'-Deoxy Adenosine | 0.38 | 0.11 | 0.88 | 0.69 | 0.96 | | * | | 0.75 | 0.84 | **1.06** | 0.07 | 0.89 | 0.15 | 0.76 | 0.40 |  |
| 3-Methyl-D-Glucose | 0.37 | 0.82 | 0.89 | 0.44 | 0.74 | | 0.71 | | 0.77 | 0.69 | 0.85 | 0.87 | 0.85 | 0.39 | 0.78 | 0.42 |  |
| Acetic Acid | 0.66 | ** | 0.92 | 0.29 | 0.76 | | 0.58 | | 0.87 | 0.17 | **2.01** | * | 0.94 | * | 0.91 | * |  |
| Adenosine | 0.37 | 0.40 | 0.96 | 0.24 | 0.83 | | 0.34 | | 0.75 | 0.82 | 0.88 | 0.38 | 0.89 | 0.31 | 0.76 | 0.83 |  |
| Adenosine-5'-Monophosphate | 0.39 | 0.26 | 0.86 | 0.63 | 0.78 | | 0.28 | | 0.80 | 0.65 | 0.85 | 0.55 | 0.90 | 0.21 | 0.80 | 0.67 |  |
| alpha-Cyclodextrin | 0.37 | 0.19 | 0.83 | 0.83 | 0.84 | | 0.13 | | 0.77 | 0.93 | 0.85 | 0.87 | 0.82 | 0.62 | 0.84 | * |  |
| alpha-D-Glucose | 0.63 | ** | **1.12** | * | 0.81 | | 0.12 | | 0.87 | 0.74 | **1.43** | ** | 0.87 | 0.24 | 0.90 | * |  |
| alpha-D-Glucose-1-Phosphate | 0.37 | 0.40 | 0.93 | 0.29 | 0.82 | | 0.17 | | 0.75 | 0.69 | **1.12** | 0.18 | 0.82 | 0.62 | 0.74 | 0.64 |  |
| alpha-D-Lactose | 0.44 | ** | 0.99 | 0.23 | 0.75 | | 0.50 | | 0.87 | 0.18 | **1.65** | ** | 0.88 | 0.19 | 0.83 | * |  |
| alpha-Hydroxybutiric Acid | 0.66 | ** | 0.87 | 0.73 | **1.14** | | ****** | | 0.74 | 0.91 | **1.05** | 0.07 | **1.09** | ** | **1.04** | ** |  |
| alpha-Ketoglutaric Acid | 0.47 | ** | 0.96 | 0.26 | 0.86 | | 0.05 | | **1.20** | 0.10 | **1.85** | ** | 0.94 | * | 0.79 | 0.19 |  |
| alpha-Ketovaleric Acid | 0.39 | 0.20 | 0.86 | 0.51 | 0.75 | | 0.40 | | 0.75 | 0.72 | **1.03** | 0.24 | 0.90 | 0.07 | 0.77 | 0.32 |  |
| alpha-Methyl-D-Galactoside | 0.61 | ** | 0.92 | 0.29 | 0.86 | | 0.25 | | **2.00** | * | **1.74** | * | 0.82 | 0.56 | **1.41** | ** |  |
| alpha-Methyl-D-Glucoside | 0.45 | * | 0.97 | 0.22 | 0.78 | | 0.19 | | **1.06** | 0.15 | 0.99 | 0.17 | 0.93 | * | 0.96 | ** |  |
| alpha-Methyl-D-Mannoside | 0.52 | ** | 0.97 | 0.26 | 0.84 | | 0.12 | | 0.86 | 0.13 | **1.10** | 0.11 | 0.98 | * | **1.59** | ** |  |
| Amygdalin | 0.55 | ** | 0.96 | 0.39 | 0.85 | | 0.08 | | **1.30** | 0.07 | **1.07** | 0.23 | 0.89 | 0.23 | 0.84 | * |  |
| Arbutin | 0.53 | ** | 0.97 | 0.32 | 0.74 | | 0.54 | | 0.98 | 0.21 | **1.18** | * | 0.85 | 0.44 | **1.16** | ** |  |
| beta-Cyclodextrin | 0.44 | ** | 0.86 | 0.23 | 0.90 | | * | | 0.76 | 0.64 | 0.88 | 0.43 | 0.92 | 0.11 | **1.23** | ** |  |
| beta-Hydroxybutiric Acid | 0.63 | ** | 0.91 | 0.31 | 0.77 | | 0.32 | | 0.76 | 0.34 | **1.36** | * | 0.90 | 0.12 | **1.14** | ** |  |
| beta-Methyl-D-Galactoside | 0.45 | * | 0.92 | 0.17 | 0.87 | | 0.15 | | **1.33** | * | **1.38** | * | **1.07** | * | **1.34** | ** |  |
| beta-Methyl-D-Glucoside | 0.49 | ** | 1.00 | 0.24 | 0.92 | | 0.10 | | **1.21** | 0.06 | **1.34** | * | 0.89 | 0.31 | **1.14** | ** |  |
| D-Alanine | 0.47 | * | 0.94 | 0.28 | 0.86 | | 0.15 | | 0.84 | 0.38 | 0.95 | 0.35 | 0.92 | 0.19 | 0.98 | * |  |
| D-Arabitol | 0.70 | ** | 0.92 | 0.47 | 0.81 | | 0.17 | | 0.89 | 0.14 | **2.29** | ** | 0.88 | 0.27 | 0.89 | * |  |
| D-Cellobiose | 0.59 | ** | **1.10** | 0.06 | 0.79 | | 0.18 | | **1.79** | * | **1.28** | * | 0.87 | 0.33 | 0.84 | 0.10 |  |
| D-Fructose | 0.47 | ** | **1.03** | 0.10 | 0.85 | | * | | **1.02** | 0.46 | **1.12** | 0.06 | 0.97 | * | 0.83 | * |  |
| D-Fructose-6-Phosphate | 0.34 | 0.53 | 0.87 | 0.24 | 0.81 | | 0.27 | | **1.14** | 0.12 | **1.29** | * | 0.95 | 0.10 | 0.76 | 0.57 |  |
| D-Galactose | 0.60 | ** | **1.10** | 0.11 | 0.79 | | 0.22 | | **1.51** | 0.20 | **1.23** | * | **1.00** | * | 0.85 | 0.12 |  |
| D-Galacturonic Acid | 0.53 | ** | **1.03** | 0.11 | 0.81 | | 0.15 | | **1.73** | * | **1.37** | 0.06 | 0.95 | * | 0.94 | * |  |
| D-Gluconic Acid | 0.68 | ** | 0.92 | 0.66 | 0.90 | | 0.10 | | **1.09** | 0.10 | **2.04** | ** | 0.80 | 0.59 | **1.35** | ** |  |
| D-Glucose-6-Phosphate | 0.33 | 0.74 | 0.89 | 0.31 | 0.77 | | 0.40 | | 0.78 | 0.43 | **1.19** | * | 0.85 | 0.42 | 0.77 | 0.45 |  |
| D-L-alpha-Glycerol Phosphate | 0.31 | 0.83 | 0.84 | 0.78 | 0.84 | | 0.18 | | **1.02** | 0.14 | 0.97 | 0.72 | 0.77 | 0.68 | 0.78 | 0.50 |  |
| D-Lactic Acid Methyl Ester | 0.57 | ** | 0.95 | 0.25 | 0.92 | | 0.06 | | 0.93 | 0.19 | **1.80** | * | **1.00** | * | **1.09** | ** |  |
| D-Malic Acid | 0.78 | ** | 0.88 | 0.44 | 0.93 | | 0.07 | | 0.98 | 0.21 | **1.48** | 0.11 | 0.91 | 0.21 | **1.30** | ** |  |
| D-Mannitol | 0.62 | ** | 0.98 | 0.20 | 0.82 | | 0.09 | | **1.60** | * | **1.91** | * | 0.88 | 0.21 | 0.82 | 0.09 |  |
| D-Mannose | 0.64 | ** | **1.04** | 0.10 | 0.80 | | 0.12 | | **1.26** | * | **1.49** | * | 0.90 | 0.13 | 0.89 | * |  |
| D-Melezitose | 0.48 | ** | **1.11** | 0.09 | 0.77 | | 0.24 | | **1.86** | * | **1.61** | ** | 0.86 | 0.40 | **1.12** | ** |  |
| D-Melibiose | 0.53 | ** | 1.00 | 0.18 | 0.89 | | ****** | | **1.20** | 0.12 | **1.71** | * | 0.98 | * | **1.06** | ** |  |
| D-Psicose | 0.43 | * | **1.02** | 0.15 | 0.87 | | 0.18 | | 0.80 | 0.33 | **1.70** | * | 0.98 | 0.23 | **1.16** | ** |  |
| D-Raffinose | 0.50 | ** | **1.08** | 0.10 | 0.88 | | * | | **1.17** | 0.09 | **1.80** | * | 0.91 | 0.10 | **1.08** | ** |  |
| D-Ribose | 0.89 | ** | 0.99 | 0.21 | **1.08** | | ****** | | **1.00** | 0.17 | **2.70** | ** | 1.13 | ** | 0.71 | 0.99 |  |
| D-Sorbitol | 0.54 | ** | 0.94 | 0.50 | 0.80 | | 0.14 | | **1.26** | * | **1.03** | 0.22 | 0.90 | 0.11 | **1.01** | ** |  |
| D-Tagatose | 0.45 | ** | 0.96 | 0.25 | 0.77 | | 0.44 | | **1.27** | * | 0.93 | 0.36 | 0.96 | * | 0.81 | 0.46 |  |
| D-Trehalose | 0.52 | ** | 0.97 | 0.38 | 0.86 | | 0.06 | | **1.53** | * | **1.36** | * | **1.06** | * | 0.84 | * |  |
| D-Xylose | 0.71 | ** | **1.03** | 0.08 | 0.83 | | 0.08 | | **1.45** | * | **1.76** | ** | 0.90 | 0.15 | 0.83 | * |  |
| Dextrin | **1.05** | ** | **1.12** | 0.10 | 0.77 | | 0.38 | | **1.21** | * | **1.64** | ** | 0.87 | 0.27 | 0.88 | 0.10 |  |
| gamma-Hydroxybutiric Acid | 0.43 | ** | 0.94 | 0.20 | 0.78 | | 0.24 | | 0.71 | 0.96 | **2.02** | ** | 0.86 | 0.37 | 0.95 | * |  |
| Gentiobiose | 0.63 | ** | **1.10** | 0.10 | 0.88 | | * | | **1.59** | * | **1.21** | * | 0.90 | 0.14 | 0.95 | * |  |
| Glycerol | 0.56 | ** | 0.91 | 0.54 | 0.74 | | 0.49 | | **2.05** | ** | **1.74** | ** | 0.86 | 0.29 | 0.70 | 1.00 |  |
| Glycogen | 0.52 | ** | **1.05** | 0.15 | 0.83 | | 0.20 | | **1.21** | 0.15 | 0.99 | 0.20 | 0.83 | 0.71 | 0.79 | 0.31 |  |
| Glycyl-L-Glutamic Acid | 0.36 | 0.39 | 0.89 | 0.20 | 0.78 | | 0.22 | | 0.77 | 0.57 | **1.55** | ** | 0.87 | 0.26 | 0.84 | 0.49 |  |
| Inosine | 0.36 | 0.16 | 0.93 | 0.31 | 0.84 | | 0.12 | | 0.99 | 0.19 | **1.36** | * | 0.91 | 0.17 | 0.70 | 0.96 |  |
| Inulin | 0.47 | ** | 0.85 | 0.83 | 0.82 | | 0.07 | | **1.47** | * | 0.90 | 0.38 | 0.98 | * | 0.95 | * |  |
| L-Alani0mide | 0.53 | ** | 0.89 | 0.47 | 0.91 | | * | | 0.76 | 0.76 | **1.64** | * | 0.94 | * | 0.78 | 0.55 |  |
| L-Alanine | 0.52 | ** | 0.95 | 0.39 | 0.82 | | 0.25 | | 0.76 | 0.53 | 0.94 | 0.37 | 0.94 | 0.13 | 0.98 | ** |  |
| L-Alanyl-Glycine | 0.56 | ** | 0.90 | 0.53 | 0.79 | | 0.16 | | 0.79 | 0.31 | 0.97 | 0.22 | 0.97 | * | 0.84 | * |  |
| L-Arabinose | 0.70 | ** | **1.02** | 0.15 | 0.79 | | 0.21 | | **1.29** | 0.10 | **1.08** | 0.10 | 0.90 | 0.13 | **1.06** | ** |  |
| L-Asparagine | 0.49 | ** | 0.93 | 0.22 | 0.88 | | * | | **2.49** | * | **1.05** | 0.08 | **1.01** | * | 0.92 | * |  |
| L-Fucose | 0.48 | ** | 0.86 | 0.44 | 0.70 | | 0.74 | | 0.76 | 0.83 | **1.15** | * | 0.82 | 0.51 | 0.79 | 0.26 |  |
| L-Glutamic Acid | 0.56 | ** | 0.89 | 0.90 | 0.86 | | * | | 0.78 | 0.45 | 0.91 | 0.43 | **1.03** | * | **1.07** | ** |  |
| L-Lactic Acid | 0.77 | ** | 0.81 | 0.91 | 0.89 | | 0.10 | | 0.75 | 0.87 | **1.52** | * | 0.97 | * | **1.57** | ** |  |
| L-Malic Acid | 0.54 | ** | 0.85 | 0.53 | 0.89 | | 0.08 | | 0.89 | 0.46 | **1.26** | 0.09 | **1.06** | ** | **1.34** | ** |  |
| L-Pyroglutamic Acid | 0.58 | ** | 0.91 | 0.24 | 0.80 | | 0.18 | | 0.87 | 0.21 | **2.03** | ** | 0.91 | 0.14 | **1.13** | ** |  |
| L-Rhamnose | 0.55 | ** | 0.98 | 0.19 | 0.78 | | 0.33 | | 0.77 | 0.50 | **1.55** | * | 0.87 | 0.28 | **1.03** | ** |  |
| L-Serine | 0.52 | ** | 0.91 | 0.78 | 0.83 | | 0.13 | | 0.78 | 0.53 | 0.86 | 0.88 | 0.98 | * | **1.07** | ** |  |
| Lactamide | 0.37 | 0.60 | 0.98 | 0.18 | 0.79 | | 0.41 | | **1.11** | * | 0.82 | 0.88 | 0.84 | 0.48 | 0.82 | 0.17 |  |
| Lactulose | 0.46 | ** | 1.00 | 0.18 | 0.74 | | 0.55 | | **1.06** | 0.12 | **1.52** | ** | 0.89 | 0.14 | 0.92 | * |  |
| m-Inositol | 0.55 | ** | 0.89 | 0.15 | 0.87 | | 0.14 | | 0.78 | 0.69 | **1.63** | * | 0.97 | 0.09 | **1.31** | ** |  |
| Maltose | 0.59 | ** | **1.20** | * | 0.86 | | 0.05 | | **1.23** | 0.11 | **2.49** | ** | 0.89 | 0.18 | **1.20** | ** |  |
| Maltotriose | 0.55 | ** | 0.99 | 0.17 | 0.81 | | 0.11 | | **1.63** | * | **1.93** | * | 0.89 | 0.15 | 0.88 | * |  |
| Man0n | 0.36 | 0.20 | 0.89 | 0.32 | 0.79 | | 0.10 | | 0.75 | 0.83 | 0.83 | 0.98 | 0.92 | * | 0.68 | 0.97 |  |
| N-Acetyl-beta-D-Mannosamine | 0.41 | 0.25 | 0.89 | 0.21 | 0.78 | | 0.33 | | 0.76 | 0.50 | **1.05** | 0.23 | 0.92 | 0.18 | 0.92 | ** |  |
| N-Acetyl-D-Glucosamine | 0.62 | ** | 0.98 | 0.15 | 0.92 | | ****** | | **1.29** | * | **1.38** | * | **1.03** | * | 0.83 | 0.06 |  |
| N-Acetyl-L-Glutamic Acid | 0.37 | 0.27 | 0.85 | 0.24 | 0.86 | | 0.19 | | 0.81 | 0.25 | 0.84 | 0.51 | 0.79 | 0.59 | 0.76 | 0.56 |  |
| p-Hydroxy-Phenylacetic Acid | 0.52 | ** | 0.88 | 0.19 | 0.86 | | 0.08 | | 0.85 | 0.25 | 0.84 | 0.92 | 0.90 | 0.16 | **1.18** | ** |  |
| Palatinose | 0.48 | ** | **1.08** | 0.09 | 0.81 | | 0.12 | | **1.31** | 0.07 | **1.91** | ** | 0.93 | * | **1.11** | ** |  |
| Propionic Acid | 0.64 | ** | 0.87 | 0.73 | 0.82 | | 0.14 | | **1.91** | * | 0.85 | 0.21 | **1.00** | * | 0.98 | ** |  |
| Putrescine | 0.53 | ** | 0.89 | 0.45 | 0.85 | | 0.14 | | 0.76 | 0.82 | **1.73** | ** | 0.94 | 0.08 | **1.14** | ** |  |
| Pyruvic Acid | 0.71 | ** | 0.98 | 0.16 | 0.81 | | 0.21 | | **2.19** | * | **2.26** | ** | 0.89 | 0.11 | **1.26** | ** |  |
| Pyruvic Acid Methyl Ester | 0.55 | ** | 0.95 | 0.40 | 0.74 | | 0.51 | | **1.00** | 0.20 | **2.03** | ** | 0.89 | 0.18 | **1.14** | ** |  |
| Salicin | 0.56 | ** | **1.12** | * | 0.76 | | 0.42 | | **1.03** | 0.89 | **1.45** | * | 0.84 | 0.48 | 0.80 | 0.35 |  |
| Sedoheptulosan | 0.51 | ** | 0.95 | 0.27 | 0.91 | | 0.06 | | 0.76 | 0.65 | 0.88 | 0.24 | 0.91 | 0.10 | **1.37** | ** |  |
| Stachyose | 0.50 | ** | 1.00 | 0.14 | 0.73 | | 0.57 | | 0.75 | 0.86 | **1.76** | ** | 0.84 | 0.50 | **1.23** | ** |  |
| Succi0mic Acid | 0.49 | * | 0.93 | 0.32 | 0.81 | | 0.12 | | 0.75 | 0.86 | 0.82 | 0.84 | 0.90 | 0.09 | 0.99 | ** |  |
| Succinic Acid | 0.45 | * | 0.96 | 0.26 | 0.94 | | * | | 0.77 | 0.49 | 0.87 | 0.75 | 0.99 | * | **1.22** | ** |  |
| Succinic Acid Mono-Methyl Ester | 0.68 | ** | 1.00 | 0.17 | 0.80 | | 0.18 | | 0.74 | 0.96 | 0.88 | 0.73 | 0.99 | * | **1.70** | ** |  |
| Sucrose | 0.53 | ** | **1.06** | 0.14 | 0.82 | | 0.13 | | **1.16** | 0.18 | **1.98** | ** | 0.89 | 0.14 | **1.00** | ** |  |
| Thymidine | 0.37 | 0.30 | 0.94 | 0.18 | 0.80 | | 0.30 | | **1.20** | * | 0.84 | 0.84 | 0.94 | 0.18 | 0.78 | 0.38 |  |
| Thymidine-5'-Monophosphate | **1.01** | ** | 0.98 | 0.15 | 0.80 | | 0.14 | | **1.31** | * | 0.83 | 0.94 | 0.87 | 0.24 | 0.75 | 0.83 |  |
| Turanose | 0.44 | ** | **1.05** | 0.16 | 0.83 | | 0.14 | | **1.01** | 0.36 | **1.03** | 0.22 | 0.97 | * | **1.22** | ** |  |
| Tween 40 | 0.87 | ** | **1.17** | * | 0.79 | | 0.18 | | **3.01** | ** | **1.28** | * | 0.87 | 0.24 | 0.98 | * |  |
| Tween 80 | 0.63 | ** | **1.06** | 0.12 | 0.79 | | 0.19 | | **2.64** | * | **1.28** | * | 0.93 | * | 0.78 | 0.24 |  |
| Uridine | 0.36 | 0.26 | 0.94 | 0.35 | 0.75 | | 0.52 | | **1.35** | * | 0.86 | 0.43 | 0.89 | 0.09 | 0.81 | 0.70 |  |
| Uridine-5'-Monophosphate | 0.35 | 0.34 | 0.95 | 0.53 | 0.74 | | 0.57 | | **1.04** | 0.11 | 0.93 | 0.38 | 0.81 | 0.83 | 0.83 | 0.73 |  |
| Xylitol | 0.36 | 0.45 | 0.95 | 0.14 | 0.83 | | 0.22 | | 0.84 | 0.64 | **1.15** | * | 0.88 | 0.28 | 0.79 | 0.36 |  |
| **Total number of resources for which K>1** | | 2 | | 24 | | 2 | | | | 64 | | 35 | | 44 | | 66 | |
| **Total number of resources for P<0.05** | | 74 | | 4 | | 13 | | | | 75 | | 58 | | 26 | | 52 | |
